# Bacterial growth and ceftriaxone activity in individual ascitic fluids in an *in vitro* model of spontaneous bacterial peritonitis

**DOI:** 10.3389/fphar.2023.1124821

**Published:** 2023-03-29

**Authors:** Wisse van Os, Beatrix Wulkersdorfer, Sabine Eberl, Zoe Oesterreicher, Philipp Schwabl, Thomas Reiberger, Rafael Paternostro, Maria Weber, Birgit Willinger, Markus Zeitlinger

**Affiliations:** ^1^ Department of Clinical Pharmacology, Medical University of Vienna, Vienna, Austria; ^2^ Department of Internal Medicine I, Division of Infectious Diseases and Tropical Medicine, Medical University of Vienna, Vienna, Austria; ^3^ Department of Internal Medicine II, Division of Gastroenterology and Hepatology, Medical University of Vienna, Vienna, Austria; ^4^ Department of Microbiology, Medical University of Vienna, Vienna, Austria

**Keywords:** ascites, ascitic fluid, bacterial growth, ceftriaxone, spontaneous bacterial peritonitis, time-kill, pharmacokinetics-pharmacodynamics, target site

## Abstract

**Introduction:** The environment of the infection site affects bacterial growth and antibiotic activity. When bacterial growth and antibiotic activity are studied in body fluids, samples of multiple subjects are usually pooled, averaging out potentially relevant differences in composition. The ascitic fluid (AF) environment is frequently associated with spontaneous bacterial peritonitis (SBP) in cirrhotic patients. In this study, bacterial growth and ceftriaxone activity were evaluated in individual AF using an *in vitro* model of SBP, reflecting the environment and pharmacokinetics at the infection site.

**Methods:** AF was obtained from nine cirrhotic patients with non-infected ascites. Growth of nine bacterial strains (three *Escherichia coli*, four *Staphylococcus aureus*, one *Enterococcus faecalis*, and one *Klebsiella pneumoniae*) in individual AF was assessed and correlated with biomarkers including potential risk factors for SBP. Ceftriaxone time-kill experiments, in which the pharmacokinetic profile observed in AF following a 1 g intravenous infusion was replicated, were performed with two *E. coli* and two *S. aureus* isolates with minimum inhibitory concentrations around the ceftriaxone resistance breakpoint.

**Results:** Significant correlations were found between bacterial growth and AF levels of protein (Spearman’s rank correlation coefficient ρ = −0.35), albumin (ρ = −0.31), and complement C3c (ρ = −0.28), and serum levels of bilirubin (ρ = 0.39) and aspartate aminotransferase (ρ = 0.25). Ceftriaxone was active in AF, even against resistant isolates, generally resulting in ≥2 log reductions in bacterial count within 24 h.

**Conclusion:** Ascites patients may be predisposed to or protected against SBP based on the antimicrobial capacity of their AF. Ceftriaxone at clinical AF concentrations is active in the AF environment.

## 1 Introduction

Ascites is the accumulation of fluid in the peritoneal cavity, most commonly as a consequence of cirrhosis. Hospitalized patients with cirrhosis and ascites frequently develop spontaneous bacterial peritonitis (SBP), an infection of the ascitic fluid (AF), with reported incidence rates between 7% and 30% (Marciano et al., 2019). SBP is associated with poor clinical outcomes, with overall mortality rates of 32.5% and 66.2% at 1 and 12 months, respectively (Arvaniti et al., 2010). Upon diagnosis, empirical antibiotic treatment should be initiated immediately to prevent sepsis (European Association for the Study of the Liver, 2018). However, few data are reported on the activity of antibiotics in the AF environment (Miglioli et al., 2005), and studies using clinical endpoints are challenging in patients with SBP.

Microbiological *in vitro* studies are generally performed using nutrient media. This has advantages related to standardization and reproducibility but ignores potential effects of host factors on bacterial growth and antibiotic activity. Studies have found altered antibiotic activity when substituting standardized growth media for body fluids, such as urine (Erdogan-Yildirim et al., 2011; Yang et al., 2014), bile (Rees and Elliott, 1998; Wulkersdorfer et al., 2017), cerebrospinal fluid (Matzneller et al., 2016), peritoneal fluid (König et al., 1998; Miglioli et al., 1998) or, indeed, AF (Miglioli et al., 2005). However, data on bacterial growth and antibiotic activity in body fluids remains scarce (Nussbaumer-Pröll and Zeitlinger, 2020). Moreover, samples of multiple subjects are often pooled, averaging out potentially relevant differences in composition. The use of static and sometimes clinically irrelevant drug concentrations further limits the translational relevance of many *in vitro* experiments.

We aimed to establish an *in vitro* model of SBP, reflecting both the environment and pharmacokinetics at the infection site. Using the model, we assessed bacterial growth and the activity of ceftriaxone, a recommended option for prophylaxis and treatment of community-acquired SBP (European Association for the Study of the Liver, 2018; Marciano et al., 2019).

## 2 Materials and methods

### 2.1 Ascitic fluid collection and processing

AF was obtained from patients with non-infected ascites who were scheduled to undergo paracentesis. Included patients were not treated with antibiotics for at least 1 week. Patients with known HIV or hepatitis infection were excluded. Laboratory parameters were obtained for AF and matched blood samples. Aliquots of AF were cultured to confirm absence of colonization. Disk diffusion tests using *Bacillus subtilis* DSM 618 (Merck, Darmstadt, Germany) to exclude presence of antibiotic residues in the AF were performed as described previously (Wulkersdorfer et al., 2017). The remaining AF was stored at −80°C. Immediately prior to the experiments, AFs were thawed, passed through 70 μm cell strainers, and the pH was measured.

### 2.2 Bacterial growth and time-kill assays

Bacterial growth of nine strains from different species known to cause SBP (Fiore et al., 2017; Marciano et al., 2019) was evaluated in individual AF and cation-adjusted Mueller Hinton broth (CAMHB; Sigma-Aldrich, Vienna, Austria). The strains included three *Escherichia coli* strains (ATCC 25922 and two clinical isolates), four *Staphylococcus aureus* strains (ATCC 29213, ATCC 33592 and two clinical isolates), *Enterococcus faecalis* ATCC 29212 and *Klebsiella pneumoniae* ATCC 700603. Matched time-kill experiments with ceftriaxone (ceftriaxone sodium, Sigma-Aldrich, Vienna, Austria) were performed with four of the strains, due to limited availability of individual AF. Strain selection was based on the ceftriaxone minimum inhibitory concentration (MIC). MICs were determined by broth microdilution in CAMHB per guidelines from the Clinical and Laboratory Standards Institute (Clinical and Laboratory Standards Institute, 2020).

Tubes containing 5 mL individual AF or CAMHB were inoculated at a target concentration of 1.5x10^6^ colony-forming units (CFU)/mL and incubated in a shaking water bath at 37°C. In the ceftriaxone time-kill experiments, drug exposure started 1 h after inoculation. By stepwise addition of ceftriaxone at 1–3 h intervals, the concentration was gradually increased to a maximum of 12 mg/L at 8 h, replicating the clinical ceftriaxone pharmacokinetics observed in AF of cirrhotic patients following a 1 g intravenous infusion (Hary et al., 1989). At selected time points, samples were drawn after vortexing, serially diluted in duplicate in 0.9% saline and plated on Columbia agar with 5% sheep blood (bioMérieux, Marcy-l’Etoile, France). Colonies were counted after overnight incubation at 37°C. Experiments were performed in at least triplicate. For the four strains selected for time-kill experiments, data in CAMHB was obtained in sextuplicate, since not all AF experiments were conducted simultaneously.

### 2.3 Statistical analysis

The mean log-scale difference in bacterial count at the end of the experiment (24 h) compared to baseline (log_10_ ΔCFU/mL) was calculated for each combination of strain and fluid, both for the growth and ceftriaxone time-kill assays. Observations below the limit of detection (LOD; 50 CFU/mL) were imputed as LOD/2. Using Spearman’s rank order correlation, mean log_10_ ΔCFU/mL values were correlated to patient characteristics and AF and blood markers considered to be (potentially) associated with risk of developing SBP (see Table 1) (Runyon et al., 1985; Runyon, 1986; Such et al., 1988; Titó et al., 1988; Andreu et al., 1993; Guarner et al., 1999; Schwabl et al., 2015). Bonferroni correction was used to adjust for multiple testing, with an original significance level of α < 0.05. Data analysis and visualization were performed in R (R Core Team, 2020).

**TABLE 1:** Selected patient characteristics and laboratory parameters, including correlations with *in vitro* bacterial growth of nine strains in ascitic fluids of nine patients. Data are presented as mean (standard deviation) unless specified otherwise; *p* values <0.05 are indicated in bold; * marks significance at Bonferroni-adjusted α (0.0033).

		ρ	*p* value
Patient characteristics			
Aetiology of cirrhosis (ALD / cryptogenic)	6 / 3		
Child-Pugh score (B / C)	7 / 2		
Gender (% male)	100%		
Age (years)	57.2 (10.0)	0.18	0.113
Ascitic fluid parameters			
Protein (g/L)	23.6 (10.4)	−0.35	0.001*
Albumin (g/L)	13.6 (5.9)	−0.31	**0.004**
Leucocytes (G/L)	0.242 (0.148)	0.04	0.732
Complement C3c (g/L)	0.260 (0.148)^a^	−0.28	**0.010**
pH	7.46 (0.09)		
Blood parameters			
Bilirubin (μmol/L)	20.70 (9.23)	0.39	<0.001*
INR	1.4 (0.2)	0.05	0.665
AST (μkat/L)	0.756 (0.383)	0.25	**0.032**
ALT (μkat/L)	0.445 (0.244)	0.01	0.896
Albumin (g/L)	34.8 (4.0)	<0.01	0.999
Urea nitrogen (mmol/L)	5.66 (2.34)	0.06	0.608
Creatinine (μmol/L)	103.4 (28.0)	0.16	0.164
Sodium (mmol/L)	132.3 (5.4)	0.09	0.443
CRP (mg/L)	14.6 (12.1)	<0.01	0.994
Leucocytes (G/L)	6.30 (2.84)	0.10	0.385

^a^ Values of three patients were below the quantification limit (BQL, 0.2 g/L), these values were imputed as BQL/2. ρ, Spearman’s rank correlation coefficient; ALD, alcoholic liver disease; INR, International Normalized Ratio; AST, aspartate aminotransferase; ALT, alanine aminotransferase; CRP, C-reactive protein.

### 2.4 Ethics

This study was performed in accordance with the Declaration of Helsinki, European Commission good clinical practice guidelines and local good scientific practice guidelines, and approved by the ethics committee of the Medical University of Vienna (#1801/2018). Written informed consent was obtained from all patients.

## 3 Results

### 3.1 Patients

The experiments were performed with AF obtained from nine cirrhotic patients. Patient characteristics and selected AF and blood laboratory parameters are shown in Table 1. The individual parameter values are presented in Supplementary Table S1.

### 3.2 Growth assays

**FIGURE 1:**
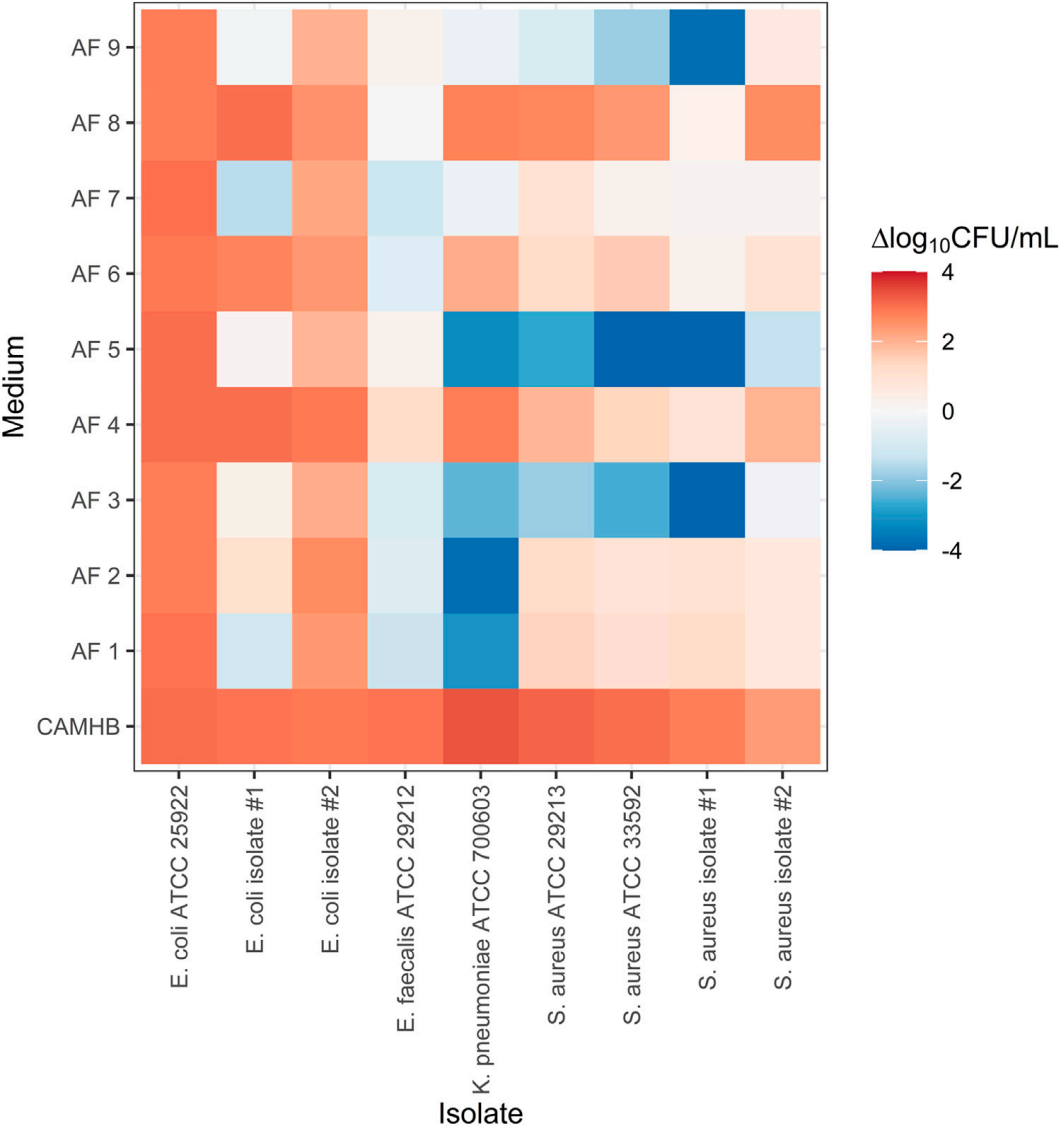
Bacterial growth of nine strains of four different species in nine individual ascitic fluids and cation-adjusted Mueller-Hinton broth. ΔCFU/mL is the mean log_10_-transformed bacterial count at the end of the experiment relative to the initial inoculum. CAMHB, cation-adjusted Mueller Hinton broth; AF, ascitic fluid; CFU, colony-forming units.

The growth experiments showed considerable variability between individual AFs (Figure 1). In some AFs, such as AF 4, AF 6 and AF 8, net bacterial growth was observed for most strains. In fluids of other patients, such as AF 3 and AF 5, approximate bacteriostasis or (sometimes extensive) reductions in bacterial count were observed for most strains. Between-strain variability was also observed. For example, bacterial concentrations of *E. coli* ATCC 25922 in all AFs were similar to those in CAMHB at 24 h, whereas net reductions were observed in six AFs for *K. pneumoniae* ATCC 700603.

### 3.3 Time-kill assays

**FIGURE 2:**
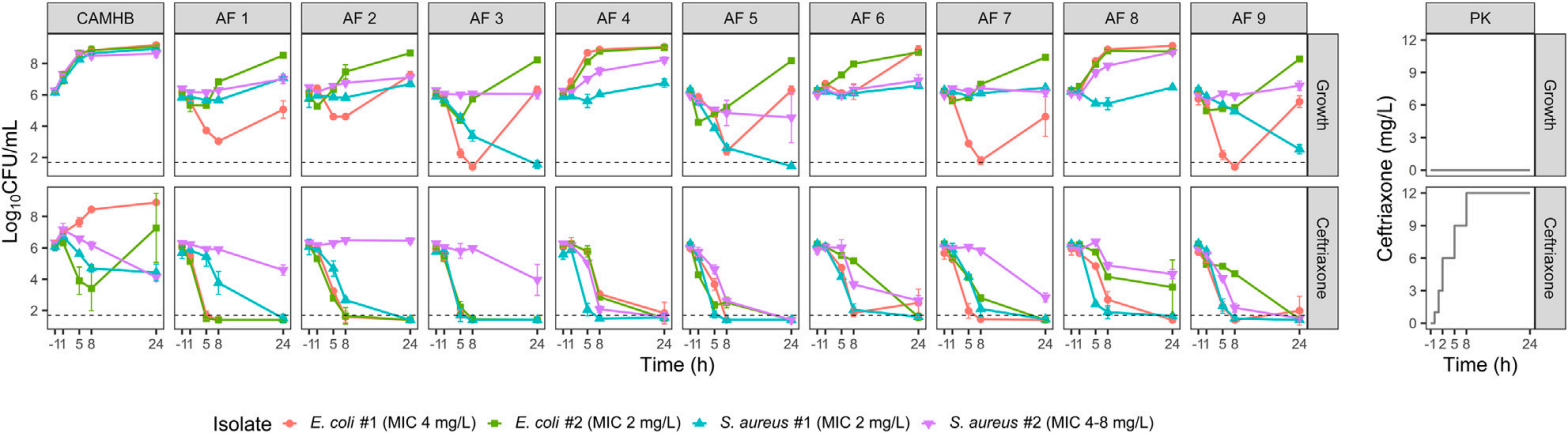
Observed bacterial counts in the growth and ceftriaxone time-kill experiments with two *E. coli* and two *S. aureus* isolates. Mean log_10_-transformed values and standard deviations are plotted. The dashed line indicates the limit of detection (LOD; 50 CFU/mL). Observations below the LOD were imputed as LOD/2. Experimental ceftriaxone concentrations are displayed in the panels on the right. CAMHB, cation-adjusted Mueller Hinton broth; AF, ascitic fluid; CFU, colony-forming units; MIC, minimum inhibitory concentration; PK, pharmacokinetics.

Two *E. coli* and two methicillin-susceptible *S. aureus* isolates were selected for the time-kill experiments (Figure 2). The MICs of *E. coli* isolates #1 and #2 and *S. aureus* isolates #1 and #2 were 4, 2, 2 and 4–8 mg/L, respectively.

**FIGURE 3:**
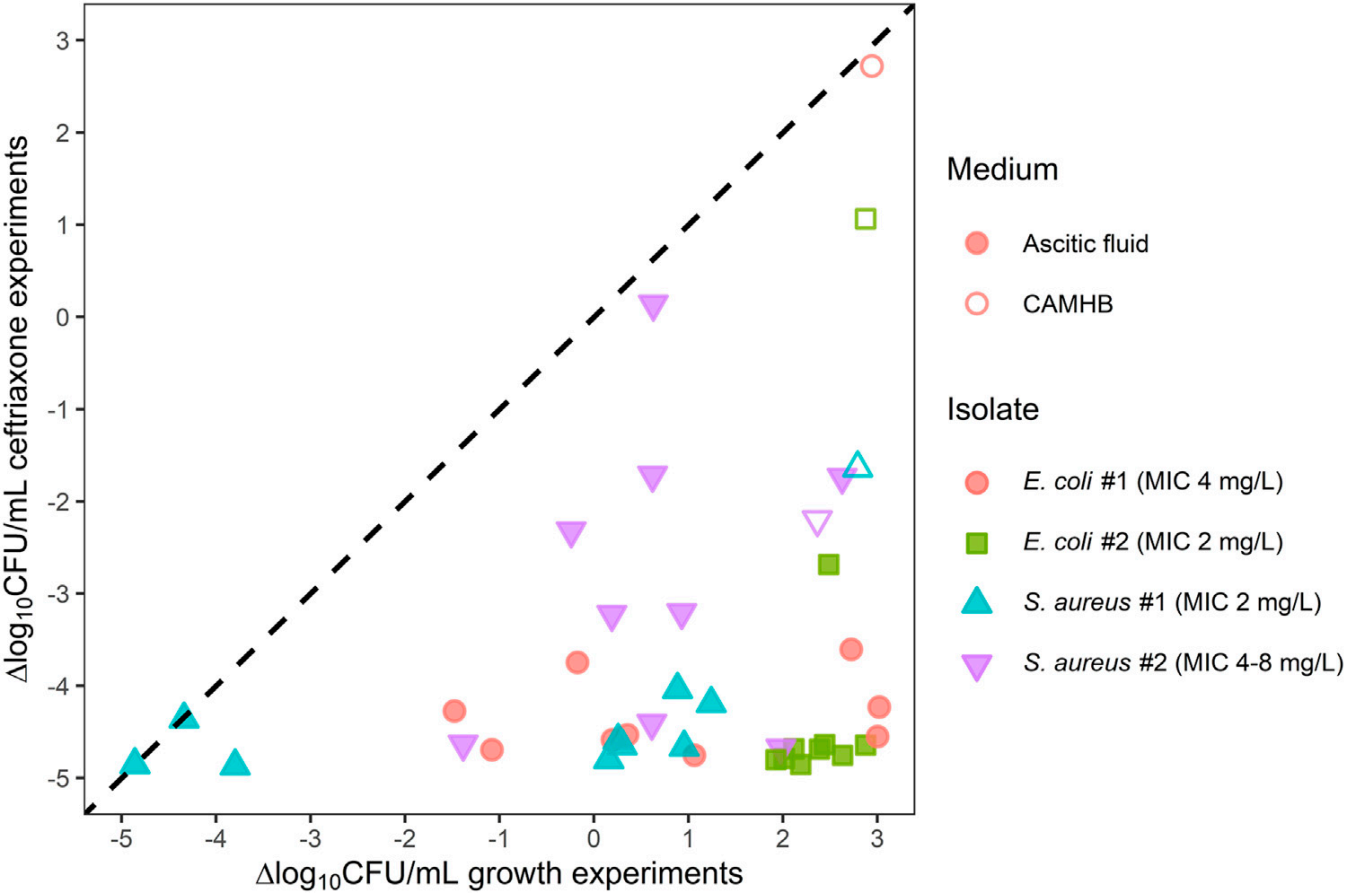
Ceftriaxone activity against two *E. coli* and two *S. aureus* isolates in individual ascitic fluids (closed symbols) or cation-adjusted Mueller Hinton broth (open symbols) plotted against growth assay results. Symbols represent the mean log_10_-transformed ΔCFU/mL for each combination of isolate and growth medium. ΔCFU/mL is the difference in bacterial count at the end of the experiment relative to the initial inoculum. Observations below the limit of detection (LOD) were imputed as LOD/2. The dashed line represents scenarios in which there is no apparent ceftriaxone activity. CAMHB, cation-adjusted Mueller Hinton broth; CFU, colony-forming units; MIC, minimum inhibitory concentration.

In AF that was not already intrinsically highly bactericidal (>2 log reduction in the growth experiments), 24 h of ceftriaxone exposure resulted in bacterial concentrations >2 log lower compared to the equivalent growth experiments (Figure 3), the only exception being *S. aureus* isolate #2 in AF 2.

In CAMHB, ceftriaxone exposure had minimal effect on *E. coli* isolate #2, and regrowth was observed for *E. coli* isolate #1. In all AFs, however, >2 log reductions compared to baseline were observed for the *E. coli* strains after 24 h of ceftriaxone exposure. Against the *S. aureus* isolates, ceftriaxone exposure in AF also generally resulted in bacterial count reductions similar to or larger than in CAMHB.

### 3.4 Correlation analyses

Significant (*p* < 0.05) negative correlations were found between log_10_ ΔCFU/mL in the growth assays and protein, albumin and complement C3c levels in AF, and significant positive correlations between log_10_ ΔCFU/mL and serum bilirubin and aspartate aminotransferase (AST) levels (Table 1). AF protein and serum bilirubin levels remained significant after adjusting for multiple testing using Bonferroni correction. The association between log_10_ ΔCFU/mL and AF pH was not tested due to the limited spread in values, with all but two AF having a pH between 7.43 and 7.50. Considering the extensive ceftriaxone effect observed in the majority of the time-kill experiments, correlations between drug effect and laboratory parameters were not tested.

## 4 Discussion

We established an *in vitro* model of SBP, reflecting both the environment and pharmacokinetics at the infection site. Importantly, we assessed bacterial growth and ceftriaxone activity in individual AF, rather than pooling samples from multiple subjects.

The observed variability in bacterial growth in antibiotic-free AF indicates that AF composition may predispose to or protect against SBP. Significant but weak negative correlations were found between bacterial growth and levels of C3c, albumin and protein in AF. These results are consistent with research indicating that complement activation, of which protein content is a surrogate marker (Runyon et al., 1985; Runyon, 1986; Such et al., 1988), is pivotal to suppress bacterial growth in AF (Fromkes et al., 1977; Simberkoff et al., 1978; Michel et al., 1980; Akalin et al., 1983; Runyon et al., 1985). The weakly positive correlations between bacterial growth in AF and serum bilirubin and AST found in our study suggest that AF of patients with impaired hepatic function better supports bacterial growth. This is in line with studies that identified increased levels of liver enzymes and/or bilirubin as risk factors for developing SBP (Titó et al., 1988; Andreu et al., 1993; Guarner et al., 1999; Schwabl et al., 2015). The correlation between hepatic function and bacterial growth *in vitro* might be reflective of the central role of the liver in producing complement proteins (Baumann et al., 2004). Our results thus support the thesis that beside general immune-suppression the increased risk of SBP in patients with impaired hepatic function can at least partly be attributed to a decreased antibacterial capacity of the AF (Simberkoff et al., 1978; Akalin et al., 1983; Andreu et al., 1993). Altogether, although the correlations were weak, they support guidelines recommending primary antibiotic prophylaxis in patients with AF protein content <15 g/L and advanced cirrhosis in order to protect those at increased risk of SBP and prevent antibiotic overuse (Fernández et al., 2016; European Association for the Study of the Liver, 2018; Marciano et al., 2019).

Due to limited availability of individual AF, ceftriaxone time-kill experiments were conducted with four isolates. Selection was based on ceftriaxone MIC, since time-kill experiments using strains with MICs much higher or lower relative to planned ceftriaxone concentrations were hypothesized to yield uninformative results. The observed bactericidal activity of ceftriaxone in AF is in line with results from Miglioli et al., which found that moxifloxacin MICs were unchanged or reduced when supplementing CAMHB with different levels of pooled AF (Miglioli et al., 2005). It should be noted that two of the isolates that were used in the time-kill experiments had ceftriaxone MICs above the clinical breakpoint of 2 mg/L (European Committee on Antimicrobial Susceptibility Testing, 2023), and would thus likely not have been treated with ceftriaxone if identified in AF through culturing and susceptibility testing. However, SBP is not diagnosed based on culture positivity but on polymorphonuclear leukocyte count, and empirical treatment should be initiated immediately after diagnosis (European Association for the Study of the Liver, 2018; Marciano et al., 2019). Our experiments reflect this empirical approach, as well as the increasing prevalence of Gram-positive and multidrug-resistant strains causing SBP (Fiore et al., 2017). The results of this study show that ceftriaxone at concentrations attained in AF following a 1 g intravenous infusion may be active against isolates with MICs above but close to the ceftriaxone breakpoint. This, and the observed bactericidal activity of ceftriaxone over 24 h against both *E. coli* isolates in AF but not in CAMHB, underlines the limitations of translating susceptibility testing in rich growth media to effect at the infection site.

A limitation of our experimental setup is the potential of nutrient depletion, especially in AF, which is likely less rich in nutrients than CAMHB. This may have affected bacterial growth. Additionally, we did not use strains isolated from patients with SBP, and used non-infected AF. Infection changes AF composition (Huang et al., 2014), and bacterial colonization in AF may not always progress to SBP, as our results also indicate. In particular the role of (increasing numbers of) leucocytes in the course of AF infection was not accounted for in our model. For these reasons, the model may be regarded as one for early-phase SBP or bacterascites, which is characterized by positive AF culture without an inflammatory response. Further, we did not study potential drug degradation in the time-kill experiments. Studies on ceftriaxone stability in aqueous solutions and serum at 37°C indicate that less than 20% of the initial concentration degrades over 24 h (Esteban et al., 1990; Cantón et al., 1993; Samara et al., 2017). However, drug degradation potentially occurs at different rates in AF and CAMHB (Wulkersdorfer et al., 2017). Finally, the extent of protein binding of ceftriaxone in AF was not measured. However, since our *in vitro* pharmacokinetic simulation was based on total drug concentrations (Hary et al., 1989), the impact of protein binding on ceftriaxone activity was automatically included in the experimental setup.

In conclusion, this study shows that AF composition affects bacterial growth and supports antibiotic prophylaxis based on AF protein content and markers indicating hepatic impairment. More clinical data are warranted to refine identification of populations at the highest risk of infection to balance benefits and risks of antibiotic prophylaxis. Moreover, these results highlight the role of the infection site environment in pharmacokinetic-pharmacodynamic relationships of antimicrobials, and shows that ceftriaxone at clinical AF concentrations is bactericidal in the AF environment.

## Data Availability

The raw data supporting the conclusion of this article will be made available by the authors, without undue reservation.
