# Supplementary material for: Bacterial growth and ceftriaxone activity in individual ascitic fluids in an in vitro model of spontaneous bacterial peritonitis
Source: Front Pharmacol. 2023 Mar 29;14:1124821. doi: 10.3389/fphar.2023.1124821 (PMC10090294; doi:10.3389/fphar.2023.1124821)
Supplement: Supplementary file 1 [file Table1.PDF]

Table S1. Selected characteristics and laboratory parameters per patient

**Parameter**	**Laboratory values related to**								
Patient characteristics	**AF 1**	**AF 2**	**AF 3**	**AF 4**	**AF 5**	**AF 6**	**AF 7**	**AF 8**	**AF 9**
Aetiology of cirrhosis	CC	ALD	ALD	ALD	ALD	CC	ALD	CC	ALD
Child-Pugh score	B	B	B	C	B	B	C	B	B
Gender	Male	Male	Male	Male	Male	Male	Male	Male	Male
Age (years)	70	65	63	57	44	68	47	56	45
Ascitic fluid parameters									
Protein (g/L)	33.9	40.3	28.3	7.1	29.5	17.7	22.1	13.8	19.7
Albumin (g/L)	20.2	22.7	15.0	5.3	19.0	11.0	11.1	8.3	10.0
Leucocytes (G/L)	0.237	0.256	0.135	0.094	NA	0.264	0.066	0.376	0.506
Complement C3c (g/L)	0.286	0.534	0.319	<0.20	0.397	0.258	0.243	<0.20	<0.20
pH	7.34	7.44	7.45	NA	7.66	7.43	7.43	7.44	7.50
Blood parameters									
Bilirubin (µmol/L)	16.2	12.5	13.9	27.5	7.4	25.3	19.5	36.8	27.2
INR	1.4	1.2	1.3	1.3	1.4	1.3	NA	1.7	1.3
AST (µkat/L)	0.25	0.60	0.50	0.85	0.50	0.84	NA	1.02	1.49
ALT (µkat/L)	0.20	0.22	0.22	0.32	0.33	0.52	0.73	0.62	0.85
Albumin (g/L)	39.8	36.4	34.6	35.8	38.7	38.0	28.9	32.0	29.2
Urea nitrogen (mmol/L)	9.82	5.07	6.36	8.82	3.96	5.82	4.61	3.18	3.32
Creatinine (µmol/L)	135.3	113.2	100.8	152.1	72.5	108.7	85.7	65.4	97.2
Sodium (mmol/L)	125	135	129	140	135	128	127	133	139
CRP (mg/L)	11.6	30.9	7.1	6.0	2.9	38.3	15.6	6.3	13.0
Leucocytes (G/L)	2.96	7.99	5.21	9.09	6.67	11.5	5.40	2.88	4.99

AF, ascitic fluid; CC, cryptogenic cirrhosis; ALD, alcoholic liver disease; NA, not available; INR, International Normalized Ratio; AST, aspartate aminotransferase; ALT, alanine aminotransferase; CRP, C-reactive protein.
